# Effect of lateral positioning on ventilation in patients with blunt thoracic injury during pressure support ventilation: the VICTORY study

**DOI:** 10.1186/s40635-026-00861-0

**Published:** 2026-02-25

**Authors:** Vorakamol Phoophiboon, Antenor Rodrigues, Matthew Ko, Luca S. Menga, Fernando Vieira, Mattia Docci, Kiana Sharifi, Annia Schreiber, Mayson L. A. Sousa, Alberto Goffi, Andrea Rigamonti, Laurent Brochard

**Affiliations:** 1 Keenan Centre for Biomedical Research, Li Ka Shing Knowledge Institute, Unity Health Toronto, 209 Victoria Street, Room 4-08, Toronto, ON M5B 1T8 Canada; 2 Interdepartmental Division of Critical Care Medicine, University of Toronto, Toronto, ON Canada; 3 Department of Critical Care, St Michael’s Hospital, Unity Health Toronto, Toronto, ON Canada; 4 Division of Critical Care Medicine, Department of Medicine, Faculty of Medicine, Chulalongkorn University, Bangkok, Thailand; 5 École de Réadaptation, Faculté de Médecine, Université Laval, Québec, QC Canada; 6 Centre de Recherche, Institut Universitaire De Cardiologie Et De Pneumologie de Québec (IUCPQ), Québec, QC Canada; 7 Fisher & Paykel Healthcare Ltd., Auckland, New Zealand; 8 Department of Medicine, University of Manitoba, Winnipeg, MB Canada

**Keywords:** Ventilation, Lateral positioning, Electrical impedance tomography, Chest trauma, Mechanical ventilation, Weaning

## Abstract

**Background:**

In patients with blunt thoracic injury requiring mechanical ventilation lateral positioning is routinely performed. Whether it modifies ventilation distribution and aeration is unclear.

**Study design and methods:**

Patients receiving pressure support ventilation (PSV) were positioned 30 degrees on each side for 30 min. Electrical impedance tomography (EIT) was used to quantify the percentage of right and left ventilation. Secondary outcomes included right and left tidal volume and the modified lung ultrasound score. At baseline, patients were categorized according to ventilation distribution: symmetrical (right lung receiving 50–55% of total ventilation) or asymmetrical.

**Results:**

Twenty-four patients were included (mean age 51 ± 20 years, 79% male) under median PSV 5 cmH_2_O [25–75% IQR: 5–8] and PEEP 8 [5–8] cmH_2_O. Trauma mechanisms included motor vehicle collision (50%) and fall (29%); 54% had bilateral rib fractures and 8% a flail chest. The duration of ventilation and ICU stay were 9 [5–19] and 13 [8–21] days, respectively.

Regional right-lung ventilation increased slightly when the lung was dependent [53% (44–58%)], decreased when non-dependent [47% (44–53%)], compared to supine [50% (45–54%)] (*p* = 0.022). These effects were observed in patients with symmetrical baseline ventilation (*n* = 8, *p* = 0.011), but not in those with asymmetrical ventilation (*n* = 16, *p* = 0.391), nor in patients with low respiratory system compliance (< 50 ml/cmH_2_O, *n* = 9, *p* = 0.539). In patients with symmetrical distribution, the right-lung and right-basal ultrasound score increased when dependent (*p* < 0.05), whereas no changes were observed in the left lung. There were no differences in respiratory mechanics or global ventilation from the beginning to the end of the session once patients were returned to supine.

**Conclusion:**

In blunt thoracic injury, lateral positioning during PSV is associated with a modest increase in regional ventilation of the dependent lung, but this effect is limited to patients with symmetrical ventilation distribution and normal compliance. In others, longer duration or higher degree of lateralization may be required.

**Supplementary Information:**

The online version contains supplementary material available at 10.1186/s40635-026-00861-0.

## Introduction

Blunt thoracic trauma is a major cause of morbidity and mortality [1–3]. In this population, factors associated with outcome include duration of mechanical ventilation (MV), intensive care unit (ICU) length of stay, and hospital-acquired infections [4–6]. Lung injury is common, yet standardized treatment guidelines aimed at improving outcomes are lacking. A recent study demonstrated possible benefit of operative management over non-operative treatment in ventilated patients with unstable chest wall injuries, although no clear benefit was observed in less severe cases [7].

Lateral body positioning is commonly performed in ICUs to promote mobilization and reduce the risk of pressure ulcers [8, 9]. Critically ill patients are frequently repositioned with the aim to mitigate the adverse effects of bed rest, inactivity, and immobilization. The potential benefits or harms of these maneuvers on lung function is unknown [8]. In mechanically ventilated ICU patients, abnormal regional distribution of ventilation, predominantly ventral and, less frequently, dorsal, can be detected using electrical impedance tomography (EIT) [10]. Such abnormalities have recently been used to predict readiness for MV separation, identify the need to reduce PEEP, and anticipate post-operative complications [10–12]. The distribution of ventilation may also differ between the right and left lungs [13–15]. These abnormalities in regional ventilation may be modifiable through positioning strategies or chest physiotherapy.

During spontaneous breathing, lateral positioning redistributes ventilation towards the dependent (“lung down”), although opposite effects have been described on end-inspiratory lung volume [16–18]. In patients with asymmetrical lung disease (i.e., pneumonia, atelectasis), previous studies have suggested that placing the healthy lung in the dependent position (i.e., lateral position) can improve oxygenation [19–23]. A “recruitment” effect induced by lateral positioning has also been suggested [18, 24]. Recently, two studies in patients with COVID-19-associated acute respiratory distress syndrome (ARDS) [25, 26] demonstrated the benefit of lateral positioning, as assessed with EIT. Lung ultrasound is a non-invasive and reproducible tool that can be used to evaluate the effect of mobilization or during weaning [27].

This prospective observational study aimed to assess whether lateral body positioning in patients with blunt thoracic injury receiving pressure support ventilation (PSV) improves regional ventilation distribution. Rotating patients to the side using a 30-degree wedge pillow is recommended to reduce pressure injury incidence and improve lung aeration, and is a common practice in many ICUs; however, its impact on lung aeration or its potential for harm has not been clearly established. To address this question, we evaluated patterns of ventilation distribution, changes in lung aeration, and other physiological parameters [28–30].

## Methods

### Study population

This study was conducted in the medical-surgical and neurotrauma ICUs of a tertiary academic critical care department in Toronto, Canada. It consecutively enrolled patients from December 2023 to October 2024. The study protocol was approved by the Unity Health Toronto Research Ethics Board (REB) at St. Michael’s Hospital approved (REB# 23-231) and registered at ClinialTrials.gov (NCT06196125). Informed consent was obtained directly from patients or their substitute decision-makers (SDMs); deferred consent was used when patients were not competent and an SDM was not immediately available.

Inclusion criteria were: age ≥ 18 years, mechanical ventilation after blunt thoracic injury with at least two rib fractures from any cause, and assisted ventilation with spontaneous breathing. Exclusion criteria included contraindications to EIT belt placement (i.e., pacemaker or defibrillator implantation, burns at the area of EIT placement), contraindications to lateral positioning (e.g., unstable spine injury), or known palliative/end-of-life status.

### Study procedures

Measurements, including EIT recording, the modified Lung Ultrasound Score (mLUSS), respiratory variables, and hemodynamic parameters, were obtained with patients in a semi-recumbent position (30 degrees head-of-bed elevation) in the following sequence:

1. Supine (baseline) for 5 min; patients had remained supine for more than 30 min before baseline measurements.
2. Lateral position, 1st side down (using a 30-degree wedge pillow) for 30 min
3. Lateral position, 2nd side down (using a 30-degree wedge pillow) for 30 min
4. Resupine for 30 min

For the first 14 patients (ID 1–13), the side with better baseline ventilation was positioned dependent (down) first; for the remaining 10 patients (ID 14–24), the opposite sequence was used (Figure E1).

MV settings were kept the same throughout the study. Clinical teams were blinded to all measurements and results.

**Fig. 1:**
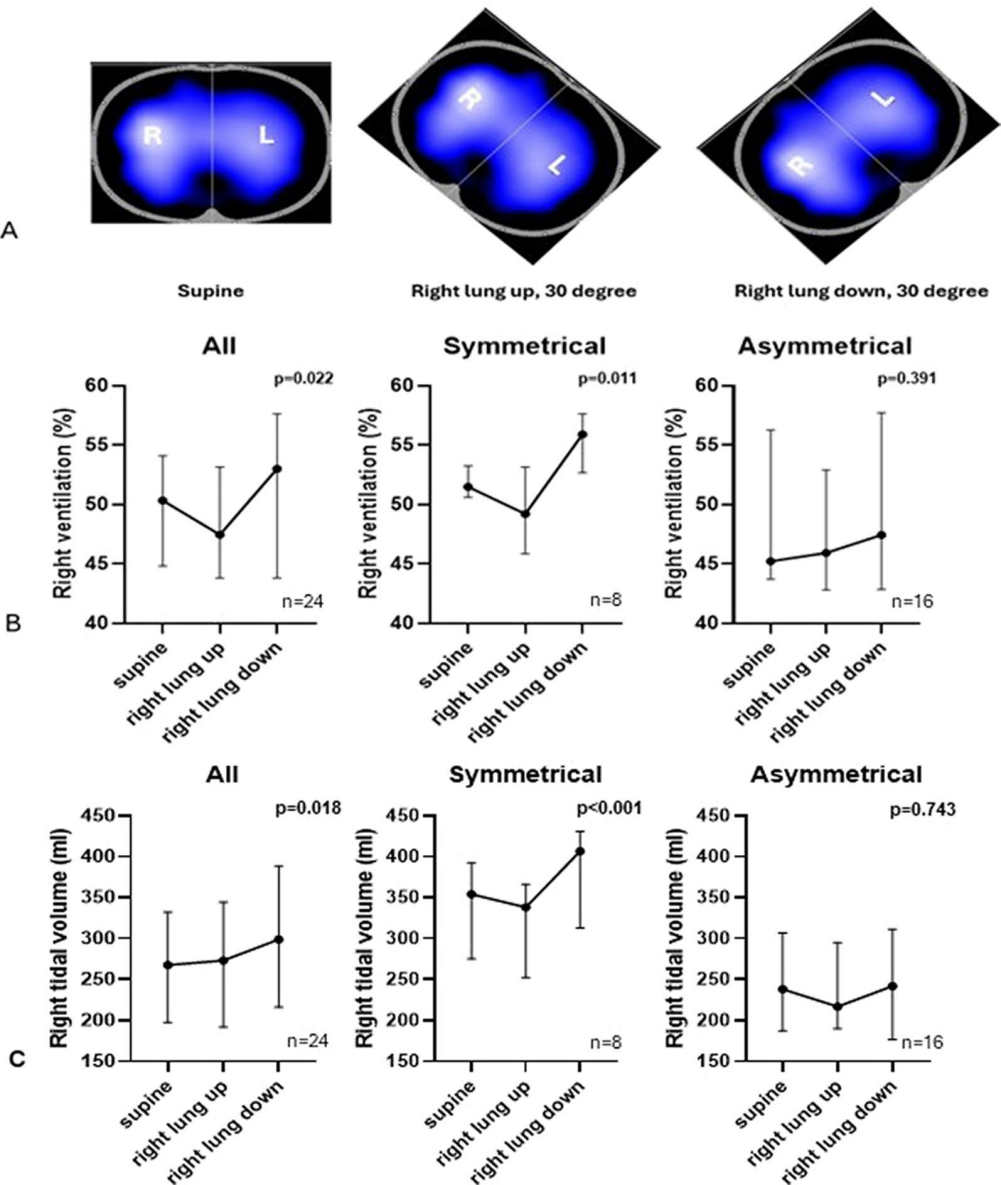
**A**. Representative examples of regional distribution of ventilation of two lungs derived from electrical impedance tomography (EIT) screenshots during 3 different positions (supine, right lung up, left lung up). **B**. Right distribution of ventilation in percentage during 3 different positions for overall (*n* = 24), symmetrical (*n* = 8) and asymmetrical groups (*n* = 16). **C**. Right volume in ml during 3 different positions for overall, symmetrical and asymmetrical groups

For analysis, each variable was compared across three different positions: supine, right lung up (and left lung down), right lung down (and left lung up), respectively (Fig. 1A).

### EIT monitoring

A 16-electrode EIT system (PulmoVista 500, Dräger Medical GmbH, Lübeck, Germany) was used to continuously monitor global and regional ventilation. The EIT belt was applied at the 4th -5th intercostal space [31], or at the 3rd-4th intercostal space when diaphragm elevation confirmed by ultrasound was likely to interfere with lung EIT assessment [32]. EIT data were analyzed offline using a dedicated software (EITdiag and EITanalysis, Dräger Medical GmbH, Lübeck, Germany). For each body position, we systematically analyzed the last period of each position, selecting a minimum of 10 consecutive artefact-free breaths. Breaths affected by coughing suctioning, or transient agitation identified at the bedside during data acquisition were excluded.

### Regional ventilation distribution and tidal volume

The tidal image displayed in real time by the EIT monitor was divided into two regions (right and left), and regional ventilation was expressed as the percentage of total tidal ventilation in each lung (R% and L%, Fig. 1A). The primary study outcome was the percentage of regional ventilation (right and left) during positional changes. Regional tidal volume for each lung was calculated as (% regional ventilation) x (global tidal volume from EIT). For simplicity, we only report right-lung ventilation % and right-lung tidal volume, as right and left values are complementary (right + left = 100%).

We initially planned to assess changes in end-expiratory lung volume (EELV); however, EELV measurements during positional changes were affected by artefacts related to patient repositioning and/or belt displacement and were therefore not considered quantitatively reliable. Changes in regional electrodes’ resistance during body positional changes led to inaccurate estimations of the impedance change at that moment.

EIT data were recorded continuously. To evaluate the temporal effect of positioning, we compared the regional distribution of ventilation and regional tidal volume of the right lung between early turning (minute 5 after repositioning) and the end of each position (last 5 min) for right lung up, right lung down and resupine. (Online Supplement).

### Ultrasound

#### Modified lung ultrasound score

We assessed lung morphology using a 2–4 MHz convex transducer to calculate the modified Lung Ultrasound Score (mLUSS) and detect differences between the two lungs. A simplified mLUSS exploring four regions per hemithorax (total score 0–24) has been proposed to simplify the technique in critically ill patients to minimize the need for repositioning [28]. The four areas of interest were: (1) antero-superior, (2) antero-inferior, (3) latero-basal, and (4) postero-basal (Figure E1).

We reported the total right and left mLUSS, as well as right and left basal mLUSS (sum of regions 3 and 4).

#### Data collection

Demographic data, comorbidities, mechanism of thoracic injury, associated injuries, thoracic injury details, severity of illness (Simplified Acute Physiology Score II, SAPS II), and Injury Severity Score (ISS) were collected at admission. At the end of each body position, we recorded the following respiratory variables: airway occlusion at 100 ms (P0.1, using pressure trigger) [33], airway pressure negative swing during an end-expiratory occlusion maneuver (ΔP_occ_) [34], inspiratory muscle pressure (Pmus; 3/4 of ΔP_occ_) [34], dynamic lung distending pressure (ΔP_L_, dyn; pressure support + (2/3 of ΔP_occ_) [34], plateau pressure during an end-inspiratory occlusion, pressure-muscular index (PMI; the difference between peak pressure and plateau pressure) [35], driving pressure (the difference between plateau pressure and positive end-expiratory pressure [PEEP]), and static respiratory compliance. Hemodynamic parameters (blood pressure, heart rate) and oxygen saturation (SpO_2_) were collected at the same time points.

#### Sample size

As no prior data were available to inform a formal sample size calculation, we planned to enroll 24 patients. This convenience sample size was based on our institutional trauma registry, which identified approximately 70 mechanically ventilated patients with at least one rib fracture per year. Given our inclusion criterion of at least two rib fractures, we estimated that approximately 24 patients would meet eligibility criteria during the study period.

#### Statistical analysis

Data were expressed as mean ± standard deviation (SD) or median [25–75% interquartile range (IQR)] according to the distribution assessed with the Shapiro–Wilk’s test. Repeated continuous measurements within the same patients across different body positions (overall and in subgroups) were analyzed using mixed-effects model with time-point (body position) as a fixed effect [36, 37]. The Holm-Šídák test was used for multiple comparison between estimated means at each position [38]. Categorical variables were compared using the chi-square test. A *p* value < 0.05 was considered statistically significant. All analyses were conducted using GraphPad Prism 10.4.2 (GraphPad Software, San Diego, CA, USA).

#### Subgroup analysis

Patients were categorized into 2 subgroups according to baseline ventilation distribution in the supine position: i. symmetrical distribution, defined as right lung receiving between 50 and 55% of total ventilation [39, 40] and ii. asymmetrical distribution, defined as right lung ventilation outside this 50–55% range (clinical characteristic in Figure E1).

We also categorized patients according to static respiratory system compliance (C_RS_) in the supine position as low (< 50 ml/cmH_2_O) or normal-to-high (≥ 50 ml/cmH_2_O) [41].

We also categorized patients according to the more ventilated (healthier) lung at supine position whether it was up or down.

## Results

### Study population

A total of 24 mechanically ventilated patients with blunt thoracic trauma and at least two rib fractures were enrolled. Characteristics of the population are summarized in Table 1. The most common mechanism of injury was motor vehicle collision (50%), followed by fall from height (29%), and post-cardiopulmonary resuscitation (21%). Thirteen (54%) patients had bilateral rib fractures, nine (38%) had unilateral rib fractures, and two (8%) had flail chest. Twenty patients (83%) had additional extra-thoracic injuries, whereas four (17%) had isolated thoracic injury.

**Table 1:** Demographic and clinical characteristics of study population and 2 subgroups

	Overall population (*n* = 24)	Symmetrical distribution of regional ventilation in two lungs (*n* = 8)	Asymmetrical distribution of regional ventilation in two lungs (*n* = 16)	*P*-value
Age, years	51 (20)	50 (17)	51 (22)	0.891
Male, *n* (%)	19 (79)	8 (100)	11 (69)	0.130
Height, cm	176 (0.1)	177 (106)	176 (96)	0.841
Body mass index, kg/m^2^	25.6 [24.1–30.2]	29.3 (6.8)	25.6 (5.1)	0.145
SAPS II	40 [30.5–47.8]	36 (19)	43 (12)	0.267
Comorbidities				
Hypertension, *n* (%)	7 (29)	3 (38)	4 (25)	0.647
Diabetes, *n* (%)	0	0	0	> 0.999
Chronic lung disease/COPD, *n* (%)	3 (13)	3 (38)	1 (6)	0.091
Coronary artery disease, *n* (%)	4 (17)	2 (25)	2 (13)	0.578
Renal failure, *n* (%)	0	0	0	> 0.999
Active cancer, n (%)	0	0	0	> 0.999
Mechanism of injury				
Motor vehicle collision, *n* (%)	12 (50)	4 (67)	8 (50)	0.646
Fall from height, *n* (%)	7 (29)	2 (33)	5 (31)	> 0.999
Post-cardiopulmonary resuscitation, *n* (%)	5 (21)	2 (33)	3 (19)	0.585
Injury Severity Score	34 (11)	39 (12)	31 (9)	0.167
Details of thoracic injury				
Unilateral rib fractures, *n* (%)	9 (38)	3 (38)	6 (38)	0.655
Bilateral rib fractures, *n* (%)	13 (54)	5 (63)	8 (50)	0.679
Flail chest, *n* (%)	2 (8)	0	2 (13)	0.536
Details of organ injury				
Isolated thoracic injuries, *n* (%)	4 (17)	2 (25)	2 (13)	0.578
Other coexisting main injuries, *n* (%)	20 (83)	6 (75)	14 (88)	0.578
- brain injury, *n* (%)	5 (21)	0	5 (31)	0.266
- abdominal injury, *n* (%)	6 (25)	1 (13)	5 (31)	0.621
- spinal cord injury, *n* (%)	2 (8)	2 (25)	0	0.101
- long bone injury, *n* (%)	7 (29)	3 (38)	4 (25)	0.647
Duration of first successful SBT to MV separation*	2 [1–3]	1 [0–2]	2 [1–4]	0.201
MV day, days	9 [5–19]	9 (7)	12 (7)	0.395
ICU LOS, days	13 [8–21]	13 (8)	16 (9)	0.544
ICU Mortality, *n* (%)	1 (4.2)	1 (17)	0	0.273
Right ventilation at supine (baseline), %	50 [45–54]	51 [51–54]	45 [44–56]	0.153

*MV separation was considered as either successful extubation or tracheostomy without using MV

*SAPS II* Simplified acute physiology score II, *COPD* Chronic obstructive pulmonary disease, *SBT* Spontaneous breathing trial, *MV* Mechanical ventilation, *ICU LOS* Intensive care unit length of stay

Continuous variables are shown as mean (SD), median [IQR] or count (percentage) as appropriate and *p*-values are from unpaired t-test or Mann–Whitney test. Categorical variables are presented as count (percentage), and *p*-values are from Chi-square or Fisher’s exact test as appropriate

MV settings and physiological measurements across the four positions are shown in Table 2 and E1 (subgroup analyses). Median pressure support was 5 [5–8] cmH_2_O, and PEEP was 8 [5–8] cmH_2_O.

**Table 2:** MV settings, respiratory and systemic variables during 3 different positions of study population

	Supine	Right lung up	Right lung down	Resupine	*P*-value
MV Settings and Respiratory Variables					
PS, cmH_2_O	8 [5–8]	8 [5–8]	8 [5–8]	8 [5–8]	NA
PEEP, cmH_2_O	8 [5–8]	8 [5–8]	8 [5–8]	8 [5–8]	NA
FiO_2_, %	35 [30–40]	35 [30–40]	35 [30–40]	35 [30–40]	NA
RR, breath/minute	19 [13–23]	18 [13–23]	18 [13–23]	20 [14–23]	0.475
P0.1, cmH_2_O	1.9 [1.2–2.6]	1.8 [1.3–2.7]	2.2 [1.1–2.9]	1.7 [1.3–2.4]	0.428
ΔPocc, cmH_2_O	10.0 [5.3–15.0]	12.3 [6.0–17.3]	10.0 [7.0–14.7]	12.0 [5.8–16.5]	0.349
Pmus, cmH_2_O	7.5 [4.0–11.3]	9.2 [4.5–12.9]	7.5 [5.3–11.0]	9.0 [4.3–12.4]	0.349
Plateau pressure, cmH_2_O	19 [15–20]	18 [15–20]	19 [15–20]	18 [15–20]	0.753
PMI, cmH_2_O	3.0 [0–6.0]	4.0 [0–6.3]	3.5 [0–6.5]	4.0 [1.0–7.8]	0.733
Driving pressure, cmH_2_O	11.0 [8.3–12.0]	10.0 [9.0–12.0]	10.5 [8.0–12.0]	10.0 [9.0–12.8]	0.785
Static respiratory system compliance, mL/cmH_2_O	53 [37–65]	51 [39–62]	51 [39–64]	50 [43–66]	0.126
ΔP_L_, dyn, cmH_2_O	12.8 [10.3–18.0]	15.5 [13.0–18.2]	12.7 [11.7–17.3]	13.8 [11.3–17.9]	0.349
SBP, mmHg	135 [123–146]	133 [124–146]	126 [119–145]	139 [130–155]	0.038
MAP, mmHg	91 [83–97]	90 [86–97]	88 [82–96]	92 [82–99]	0.304
HR, beat/minute	86 [76–97]	86 [78–105]	83 [73–101]	86 [75–116]	0.157
SpO_2_, %	97 [95–100]	99 [97–99]	98 [96–99]	98 [96–99]	0.301

*MV* Mechanical ventilation, *PS* Pressure support, *PEEP* Positive end expiratory pressure, *FiO*_*2*_ Fraction of inspired oxygen, *RR* Respiratory rate, *P0.1* Negative airway pressure generated during the first 100 ms, *ΔPocc* End-expiratory airway occlusion pressure, *Pmus* Respiratory muscle pressure, *PMI* Pressure muscle index, ΔP_L_, *dyn* Dynamic transpulmonary driving pressure, *SBP* Systolic blood pressure, *MAP* Mean arterial pressure, *HR* Heart rate, *SpO*_*2*_ Oxygen saturation

Continuous variables are shown as median [IQR]. For comparison, *p*-values were derived from mixed-effects models accounting for random subject effects. If there was no within-subject variation in the variable across conditions, the *p*-value is reported as NA (not applicable)

No change in driving pressure, plateau pressure or compliance could be observed between supine and resupine. As shown in Table 2, there were no significant differences across positions in respiratory rate, respiratory drive (P0.1), effort (ΔPocc, Pmus and PMI), driving pressure, plateau pressure, lung distending pressure (ΔP_L_, dyn), or static respiratory system compliance (C_RS_). Mean arterial pressure (MAP), heart rate, and SpO_2_ also remained unchanged. Only Systolic Blood Pressure (SBP) at supine/resupine position was slightly higher compared to the lateral position (*p* = 0.038), but this difference was not clinically relevant (Table 2).

### Regional ventilation

Because no differences were observed in regional ventilation or regional tidal volume between the initial supine and final resupine positions, the measurements during these two conditions were averaged and presented as “supine” for simplicity (Figure E4). The baseline right ventilation (%) at supine position of 2 subgroups were also not different, 51% [51–54%] versus 45% [44–56%], *p* = 0.153 (Table 1).

Right-lung regional ventilation increased when the right lung was in the dependent position and decreased when it was non-dependent, compared with supine (supine = 50% [45–54%], right lung down = 53% [44–58%], right lung up = 47% [44–53%]; *p* = 0.022). These positional effects were present in patients with symmetrical right-left distribution of ventilation at baseline (*n* = 8, supine = 51% [51–54%], right lung up = 49% [46–53%], right lung down = 56% [53–58%], *p* = 0.011), but not in those with asymmetrical distribution at baseline (*n* = 16, *p* = 0.391) (Fig. 1B).

Expressed as regional tidal volume, right-lung regional tidal volume was higher when dependent compared with supine (supine = 268 ml [209-346 ml], right lung down = 299 ml [216-389 ml], right lung up = 273 ml [192-344 ml], *p* = 0.018). In patients with symmetrical baseline distribution, right-lung regional tidal volume increased in the dependent position and decreased in the non-dependent position compared with supine (*p* < 0.001), whereas no significant changes were observed in those with asymmetrical distribution (*p* = 0.743) (Fig. 1C).

**Fig. 2:**
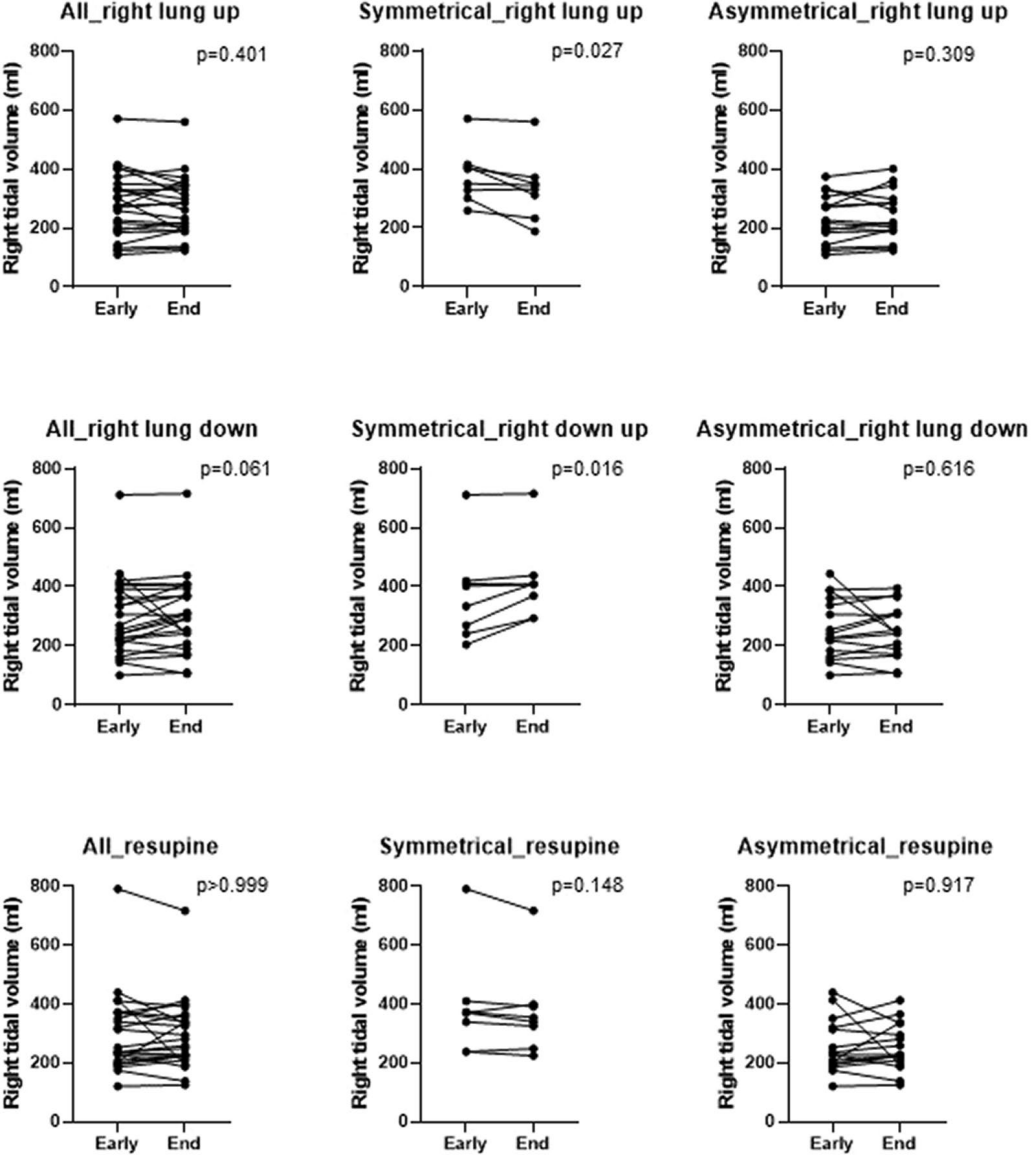
The comparison between beginning and end of the positioning session on regional tidal volume in ml of right lung during 3 different positions (right lung up, right lung down and resupine, from upper to lower row, respectively) for all, symmetrical and asymmetrical groups

Between the beginning and the end of each position regional ventilation distribution did not overall differ. In the symmetrical group, however, the right-lung regional tidal volume increased over time when the right lung was dependent (*p* = 0.016) and decreased over time when it was non-dependent (*p* = 0.027) (Fig. 2).

**Fig. 3:**
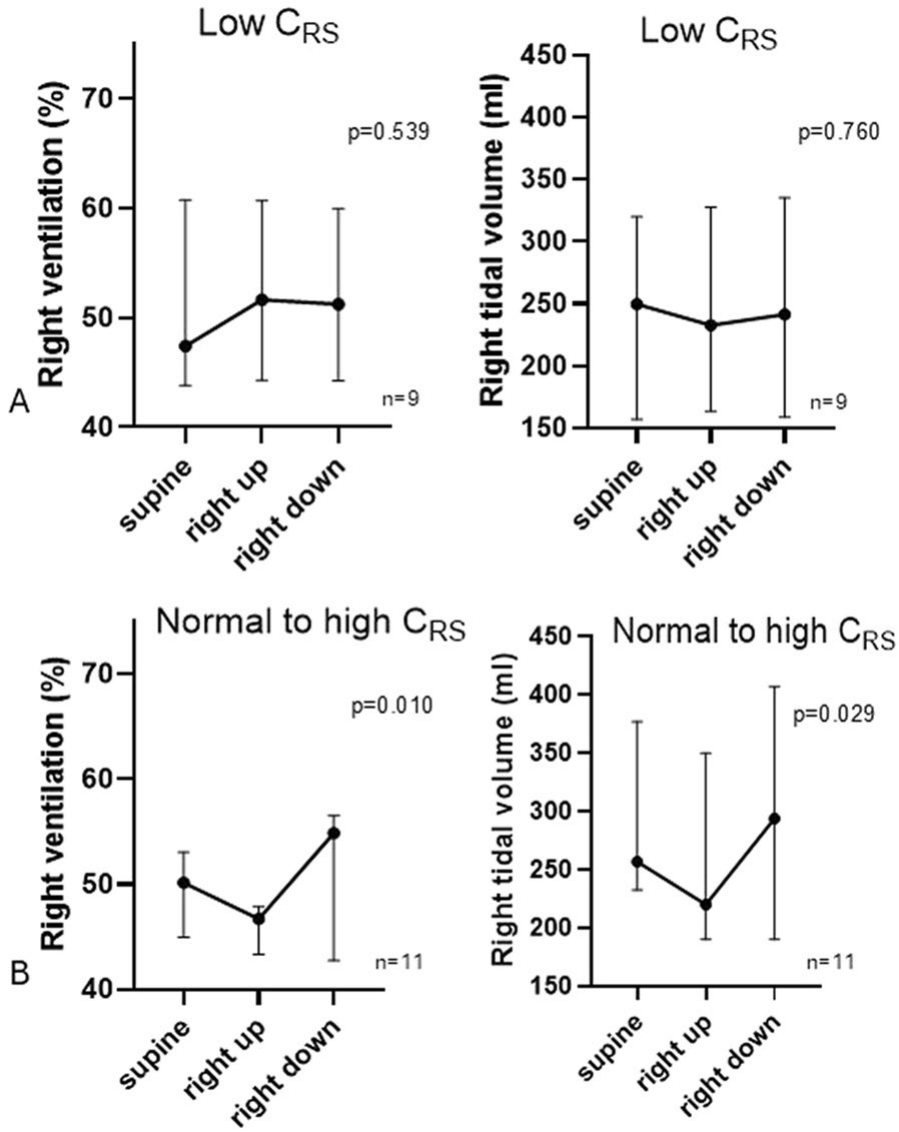
Right distribution of ventilation and right tidal volume at **A**. low static respiratory system compliance (C_RS_), *n* = 9 and **B**. normal to high C_RS_, *n* = 11. To note, plateau pressure could be measured in 20 of 24 patients in the supine position

For compliance-based analyses, plateau pressure in the supine position was available in 20 patients. Patients with low C_RS_ (*n* = 9) had a median C_RS_ of 37 [34–38] ml/cmH_2_O, whereas those with normal-to-high C_RS_ (*n* = 11) had a median C_RS_ of 63 [56–69] ml/cmH_2_O. In patients with normal-to-high C_RS_, right-lung regional ventilation (*p* = 0.010) and regional tidal volume (*p* = 0.026) increased when the lung was dependent and decreased it was non-dependent, whereas no significant changes were observed in patients with low C_RS_, (*p* = 0.539 and 0.760, respectively) (Fig. 3).

For the analyses showing the more ventilated (healthier) up or down, only patients with symmetrical ventilation at baseline demonstrated that turning the healthier lung down increased right ventilation (%) and right tidal volume and decreased when turned up, compared with the supine position (*p* = 0.040 and 0.016, respectively) (Figure E5).

### Regional mLUSS

**Fig. 4:**
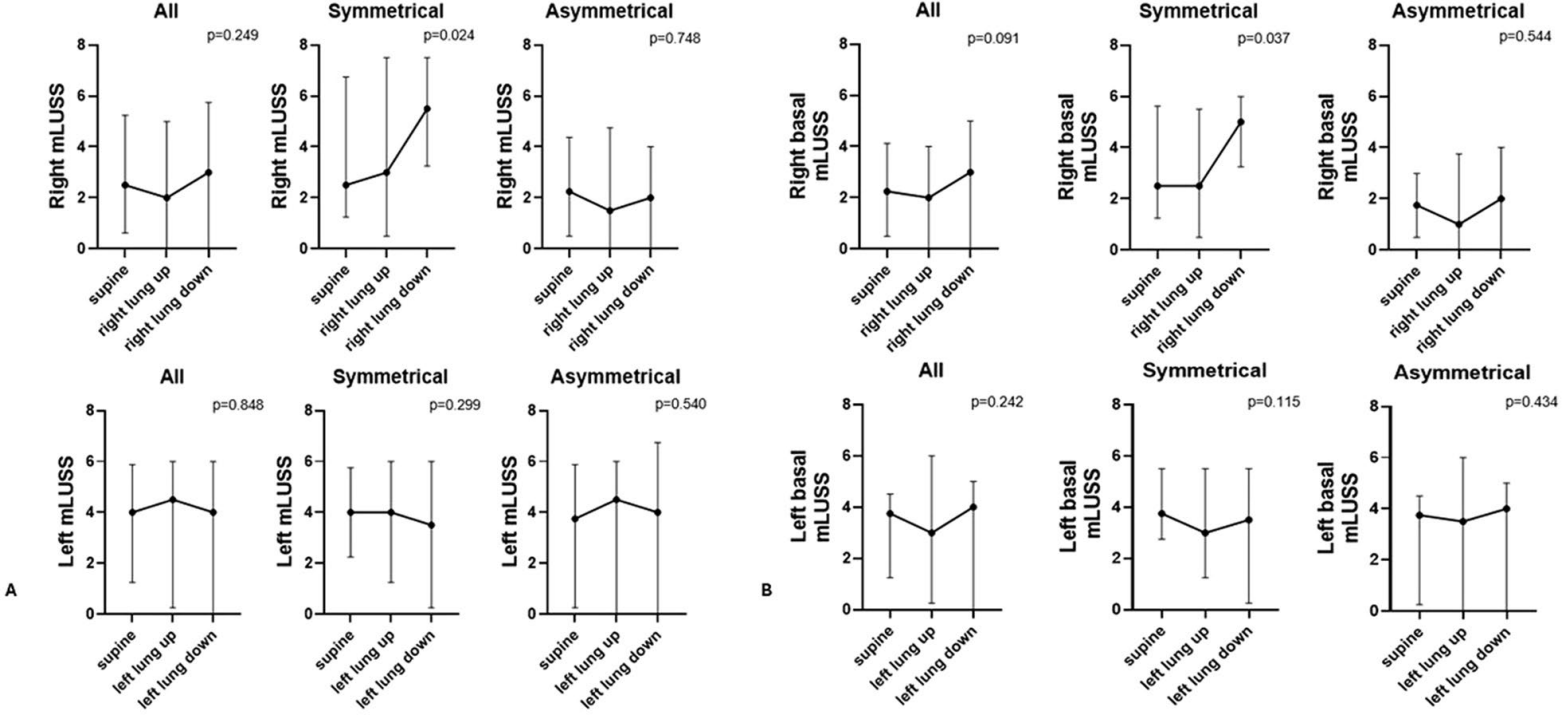
Regional mLUSS during three different positions (Right lung: supine, right lung up, right lung down; Left lung: supine, left lung up, left lung down). **A**. Right (top) and left (bottom) mLUSS for overall, symmetrical and asymmetrical groups. **B**. Right (top) and left (bottom) basal mLUSS (sum of latero-basal (area 3) and postero-basal (area 4) of mLUSS) for overall, symmetrical and asymmetrical groups

In patients with symmetrical baseline distribution, both the total right lung mLUSS and the right basal mLUSS increased (suggesting greater lung “weight”) when the right lung was dependent compared with supine (right mLUSS: 5.5 [3.3–7.5] vs. 2.5 [1.3–6.8], *p* = 0.024; right basal mLUSS: 5.0 [3.3–6.0] vs. 2.5 [1.3–5.6], *p* = 0.037). No corresponding changes were observed in the left lung (Fig. 4).

## Discussion

Our observational study provides insight into the respiratory physiological effects of routine lateral positioning in patients with blunt thoracic injury. Our results demonstrate that turning patients to 30 degrees lateral position produces modest changes in regional ventilation and tidal volume, with increased ventilation of the dependent (lung-down) lung. These effects were largely confined to patients with symmetrical ventilation at baseline or with normal-to-high respiratory system compliance. Patients with asymmetrical ventilation or low compliance showed no measurable significant effect despite a similar trend of regional ventilation changes. With respect to lung morphology, higher right lung and right basal mLUSS scores when the right lung was dependent were likewise observed only in patients with symmetrical baseline ventilation. Together, these findings suggest that routine nursing mobilization to a 30 degrees lateral position may influence regional ventilation primarily in patients with relatively preserved and symmetrical lung mechanics, without meaningful effects on respiratory drive, effort, or global compliance. In addition, lateral positioning in patients with more severe baseline lung impairment does not appear to be harmful in terms of changes in regional aerations.

In ICU patients, gravity and lung edema largely determine distribution of regional lung density, aeration, and therefore ventilation. In the lateral position, the dependent region (i.e., the part of the lung towards the bed) is expected to receive more ventilation because gravitational changes in pleural pressure reduce transpulmonary pressure gradients and favor greater tidal expansion in dependent areas [18, 24]. Our study confirms that, in patients with blunt thoracic injury, lateral positioning can increase both regional ventilation and tidal volume in the dependent lung compared with supine position, but predominantly in those with symmetrical right-left distribution, who represented a minority of our cohort.

Because the right lung is anatomically larger than left [42], we defined a “physiological” symmetrical distribution as right-lung ventilation of 50–55% (and left 45–50%), consistent with previous works [39, 40] When traumatic injury affected the lungs unequally, the right-left distribution of ventilation could deviate substantially from this range. We stratified patients into two subgroups, symmetrical (right-lung ventilation 50–55% in supine position) and asymmetrical (right-lung ventilation outside this range), to capture the spectrum of trauma-related alterations in regional ventilation. Notably, only patients with symmetrical baseline distribution demonstrated increased regional ventilation and regional tidal volume in the dependent lung with lateral positioning, whereas those with asymmetrical distribution did not change significantly.

In our study, when the lung was placed in the dependent position, we observed worse mLUSS values despite increases in regional ventilation and tidal volume observed with EIT, particularly on the right lung. This may suggest that such deterioration in regional mLUSS in dependent positions may reflect gravitational changes in lung density (i.e., increased lung weight) and not a true, contemporaneous loss of functional aeration.

Clinically, in patients with markedly asymmetrical injury, lateral decubitus positioning with the healthier lung dependent is often used to improve ventilation–perfusion matching and oxygenation. However, uncertainty persists regarding the effectiveness of lateral positioning in enhancing pulmonary gas exchange or preventing morbidity. In our cohort, respiratory drive, effort, and compliance did not change before versus after lateral positioning. By contrast, Roldan et al. [26] reported improvement in static and dynamic driving pressure and lung compliance with lateral positioning in deeply sedated and paralyzed patients with COVID-19 ARDS. This difference may relate to various factors such as lung injury severity (C_RS_ at baseline in our study 52 [39–65] vs. in COVID-19 ARDS study 34 ± 11 ml/cmH_2_O), as well as differences in sedation, paralysis and ventilatory mode. In our study, where all patients were ventilated with pressure support ventilation, lateral positioning increased regional ventilation and tidal volume in the dependent lung only in those patients with normal-to-high C_RS_, but not in those with low C_RS_.

Our study has limitations. It was conducted at a single center. The observed changes in regional distribution of ventilation and regional lung volume were relatively small and may have been influenced by the modest degree of lateral tilt (30 degrees) and the duration spent in each position. Greater degree of lateral positioning and/or longer periods may be more efficient regarding ventilation although they may be found more challenging or unsafe in patients with painful chest trauma and must be individualized. To shorten study time, patients were not returned to supine between the first and second lateral positions, which may have introduced carryover effects. We did not report EELV changes because positional shifts and variable pressure on the electrodes rendered these measurements unreliable. Finally, although the sample size was relatively small, a post hoc simulation-based power analysis indicated that the study had 79.8% power to detect an overall difference in regional ventilation distribution comparing the three body positions; more importantly there is evidence of heterogeneity of treatment effect.

## Conclusion

In patients with blunt thoracic injury receiving PSV, lateral positioning produces measurable changes in regional ventilation, but this clinically relevant impact is primarily observed in those with symmetrical baseline ventilation in supine position and normal-to-high static compliance. Respiratory drive, effort, and compliance after positioning remains unchanged with positioning. More pronounced lateral tilt and/or longer time in the lateral position may be necessary to elicit greater physiological effects.

## Supplementary Information

Additional file 1.

## Data Availability

The datasets used and/or analyzed during the current study are available from the corresponding author on reasonable request. Guarantor: Dr. Laurent Brochard.

## Abbreviations

P0.1: Airway occlusion at 100 ms
ΔP_occ_: Airway pressure negative swing during an end-expiratory occlusion maneuver
ΔP_L_, dyn: Dynamic lung distending pressure
EIT: Electrical impedance tomography
EELV: End-expiratory lung volume
ISS: Injury Severity Score
ICU: Intensive care unit
IQR: Interquartile range
MV: Mechanical ventilation
mLUSS: Modified lung ultrasound score
SpO_2_: Oxygen saturation
PEEP: Positive end-expiratory pressure
PMI: Pressure-muscular index
PS: Pressure support
Pmus: Respiratory muscle pressure
SAPS II: Simplified Acute Physiology Score II
SD: Standard deviation
C_RS_: Static respiratory system compliance
P_L_: Transpulmonary pressure
