# Supplementary material for: Effect of lateral positioning on ventilation in patients with blunt thoracic injury during pressure support ventilation: the VICTORY study
Source: Intensive Care Med Exp. 2026 Feb 25;14:20. doi: 10.1186/s40635-026-00861-0 (PMC12936264; doi:10.1186/s40635-026-00861-0)
Supplement: Supplementary file 1 — Additional file 1. [file 40635_2026_861_MOESM1_ESM.docx]

**Supplement**

**Method**

**
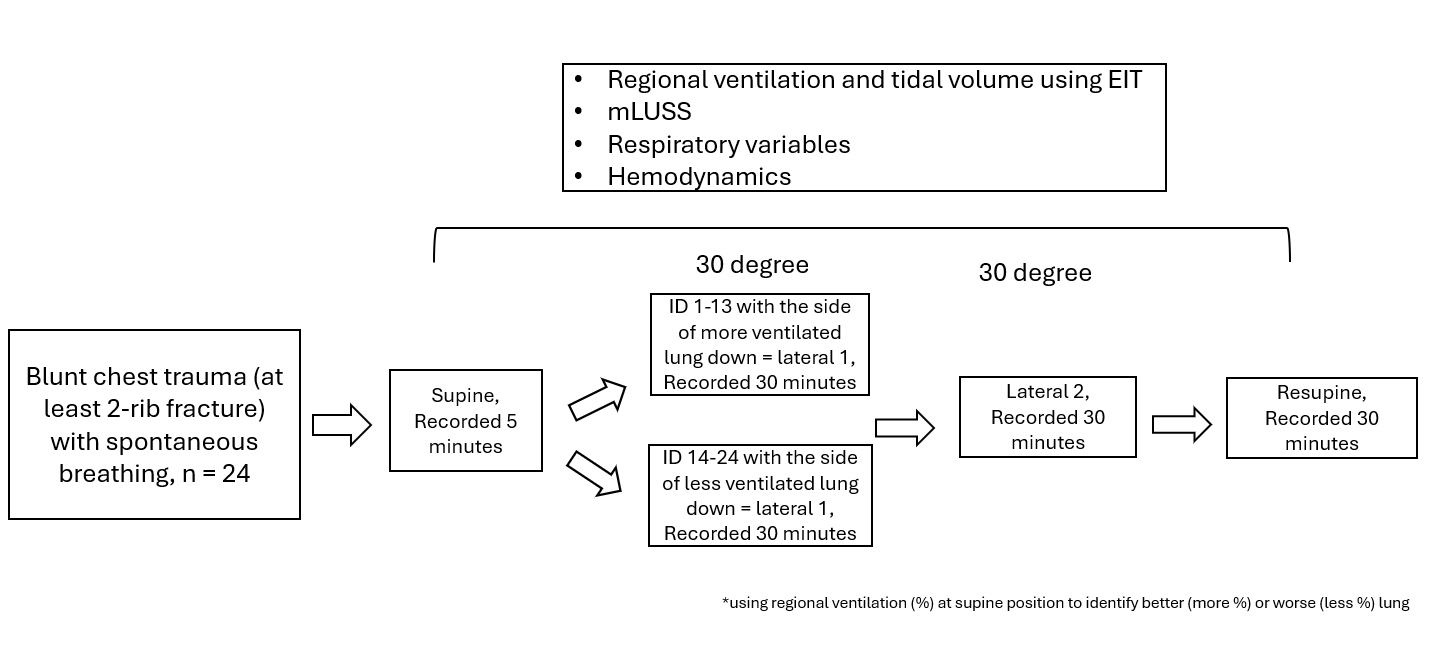
**

Figure E1. Study protocol. The first 14 patients (ID 1-13) with the side of more ventilated lung evaluated by EIT at supine position were positioned down first whereas the rest of patients (ID 14-24, n=10) with the side of less ventilated lung were positioned down first. Lateral 1 = lateral position 1^st^ side, lateral 2 = lateral position 2^nd^ side.

**
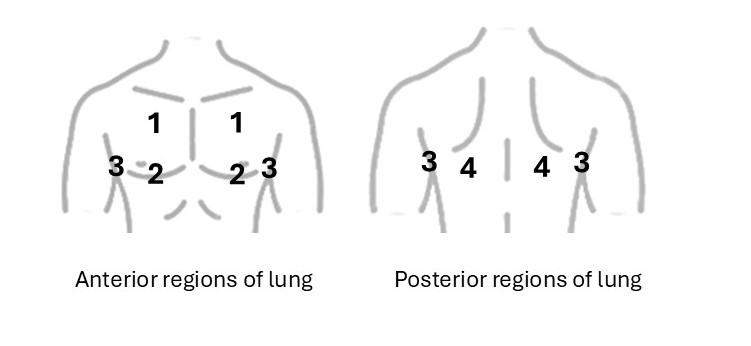
**

Figure E2. Modified lung ultrasound score (mLUSS) consisted of 4 areas at each side of the lung: 1. antero-superior, 2. antero-inferior, 3. latero-basal, 4. postero-basal.

**
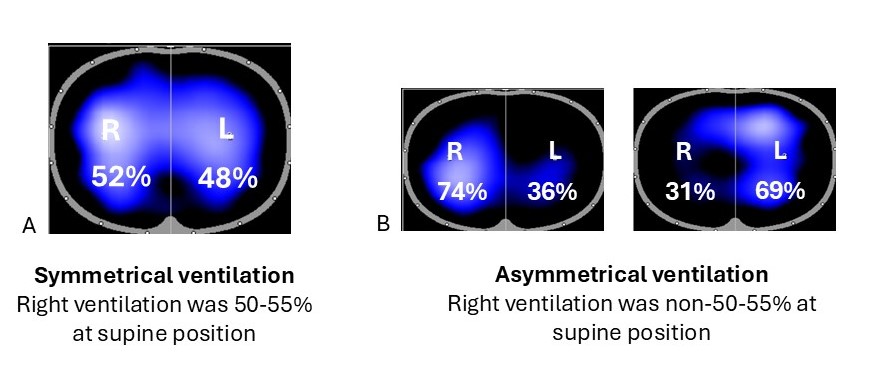
**

Figure E3. The tidal image was displayed real time on the electrical impedance tomography (EIT) screen, it was divided into 2 regions; right and left, reporting as the percentage of regional ventilation (R% and L%). A showed two lungs ventilation when they were symmetrical at supine position. B showed two lungs ventilation when they were asymmetrical at supine position.


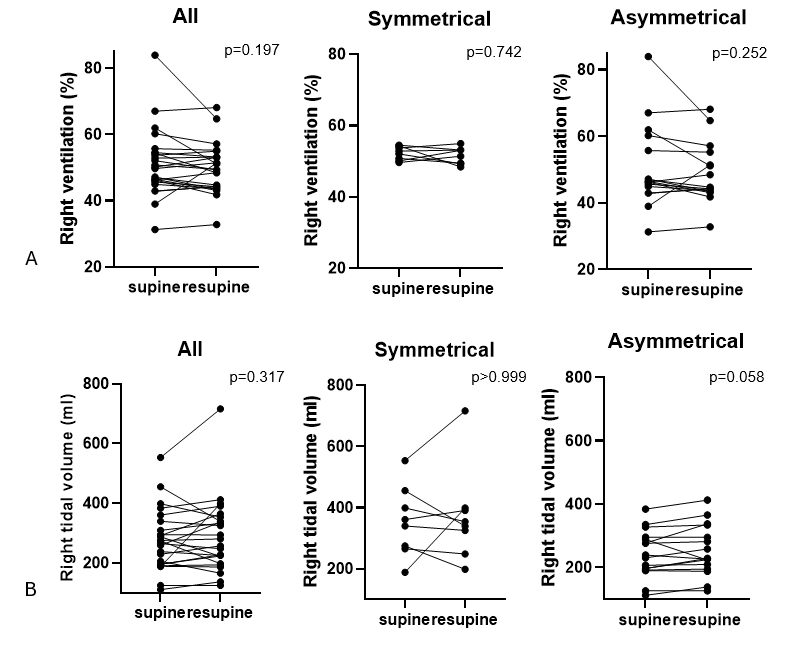


Figure E4. The comparison between supine vs resupine position of right ventilation (%), A and right tidal volume, B for overall, symmetrical and asymmetrical groups.

**Results**

Table E1. MV Settings, Respiratory and Systemic Variables of 2 Groups According the Presence of Symmetrical or Asymmetrical Patterns of Distribution of Regional Ventilation from Two Lungs during Positional Changes.

|  | Symmetrical distribution of regional ventilation in two lungs (n=8) | | | | | Asymmetrical distribution of regional ventilation in two lungs (n=16) | | | | |
| --- | --- | --- | --- | --- | --- | --- | --- | --- | --- | --- |
|  | Supine | Right lung up | Left lung up | Resupine | P-value | Supine | Right lung up | Left lung up | Resupine | P-value |
| **MV Settings and Respiratory Variables** | | | |  |  |  | | |  |  |
| PS, cmH_2_O | 8 [5-8] | 8 [5-8] | 8 [5-8] | 8 [5-8] | NA | 8 [5-8] | 8 [5-8] | 8 [5-8] | 8 [5-8] | NA |
| PEEP, cmH_2_O | 8 [5-8] | 8 [5-8] | 8 [5-8] | 8 [5-8] | NA | 8 [5-8] | 8 [5-8] | 8 [5-8] | 8 [5-8] | NA |
| FiO_2_, % | 35 [30-40] | 35 [30-40] | 35 [30-40] | 35 [30-40] | NA | 35 [30-40] | 35 [30-40] | 35 [30-40] | 35 [30-40] | NA |
| RR, breath/minute | 12 [10-19] | 11 [10-20] | 13 [8-17] | 16 [11-20] | 0.299 | 20 [16-23] | 22 [17-25] | 20 [15-23] | 22 [17-24] | 0.728 |
| P0.1, cmH_2_O | 1.7 [1.2-3.6] | 2.2 [1.5-2.7] | 2.3 [1.4-3.1] | 1.9 [1.4-3.0] | 0.696 | 2.1 [1.3-2.5] | 1.8 [1.3-2.6] | 2.2 [0.9-2.9] | 1.7 [1.0-2.3] | 0.367 |
| ΔPocc, cmH_2_O | 15.0 [12.0-18.0] | 16.0 [13.0-22.0] | 15.0 [11.0-21.0] | 16.0 [12.0-18.0] | 0.418 | 8.0 [3.0-12.0] | 10.0 [6.0-16.0] | 10.0 [6.0-10.0] | 8.0 [3.6-13.0] | 0.628 |
| Pmus, cmH_2_O | 11.3 [9.0-13.5] | 12.0 [9.8-16.5] | 11.3 [8.3-15.8] | 12.0 [9.0-13.5] | 0.418 | 6.0 [2.3-9.0] | 7.5 [4.5-12.0] | 7.5 [4.5-7.5] | 6.0 [2.7-9.8] | 0.628 |
| Plateau pressure, cmH_2_O | 20 [16-22] | 20 [17-22] | 20 [15-22] | 20 [18-21] | 0.300 | 18 [15-20] | 17 [14-20] | 18 [15-19] | 17 [15-20] | 0.491 |
| PMI, cmH_2_O | 5.0 [0-6.0] | 4.5 [0.5-9.5] | 4.5 [0.3-8.5] | 5.0 [-1.0-8.0] | 0.290 | 3.0 [-0.5-5.0] | 3.5 [0-4.5] | 3.0 [-0.8-4.0] | 3.0 [1.0-7.5] | 0.480 |
| Driving pressure, cmH_2_O | 11.0 [10.0-12.0] | 10.0 [9.3-14.5] | 11.0 [7.0-13.5] | 10.0 [9.0-13.0] | 0.324 | 11.0 [8.0-12.0] | 10.0 [8.0-12.0] | 10.5 [8.0-12.0] | 10.0 [8.5-12.5] | 0.474 |
| Static respiratory system compliance, mL/ cmH_2_O | 66 [46-74] | 61 [35-77] | 63 [43-105] | 63 [49-72] | 0.136 | 40 [36-59] | 49 [39-55] | 43 [35-54] | 50 [42-58] | 0.510 |
| ΔP_L_, dyn, cmH_2_O | 17.0 [13.0-18.3] | 17.7 [14.7-20.0] | 17.0 [12.3-20.0] | 17.0 [13.0-19.3] | 0.418 | 12.0 [9.3-16.0] | 14.0 [12.0-16.7] | 12.7 [11.7-14.7] | 13.3 [9.7-17.0] | 0.628 |
| SBP, mmHg | 134 [123-140] | 133 [123-148] | 126 [120-142] | 140 [128-148] | 0.109 | 137 [123-150] | 134 [129-146] | 133 [119-145] | 137 [130-158] | 0.251 |
| MAP, mmHg | 92 [82-97] | 92 [85-100] | 90 [86-96] | 97 [91-101] | 0.323 | 91 [83-97] | 90 [86-96] | 86 [77-96] | 86 [77-98] | 0.478 |
| HR, beat/minute | 84 [75-91] | 82 [78-88] | 84 [75-92] | 82 [73-95] | 0.449 | 89 [76-107] | 87 [78-109] | 81 [62-103] | 91 [75-122] | 0.196 |
| SpO_2_, % | 97 [96-97] | 99 [97-99] | 98 [97-100] | 98 [96-98] | 0.091 | 98 [95-100] | 99 [96-100] | 98 [95-99] | 98 [95-99] | 0.597 |

Definition of abbreviations: MV = mechanical ventilation, PS = pressure support, PEEP = positive end expiratory pressure, FiO_2_ = fraction of inspired oxygen, RR = respiratory rate, P0.1 = negative airway pressure generated during the first 100 millisecond, ΔPocc = end-expiratory airway occlusion pressure, Pmus = respiratory muscle pressure, PMI = pressure muscle index, ΔP_L_, dyn = dynamic transpulmonary driving pressure, SBP = systolic blood pressure, MAP = mean arterial pressure, HR = heart rate, SpO_2_ = oxygen saturation

Notes: Continuous variables are shown as median [IQR]. For comparison, p-values were derived from mixed-effects models accounting for random subject effects. If there was no within-subject variation in the variable across conditions, the p-value is reported as NA (not applicable).


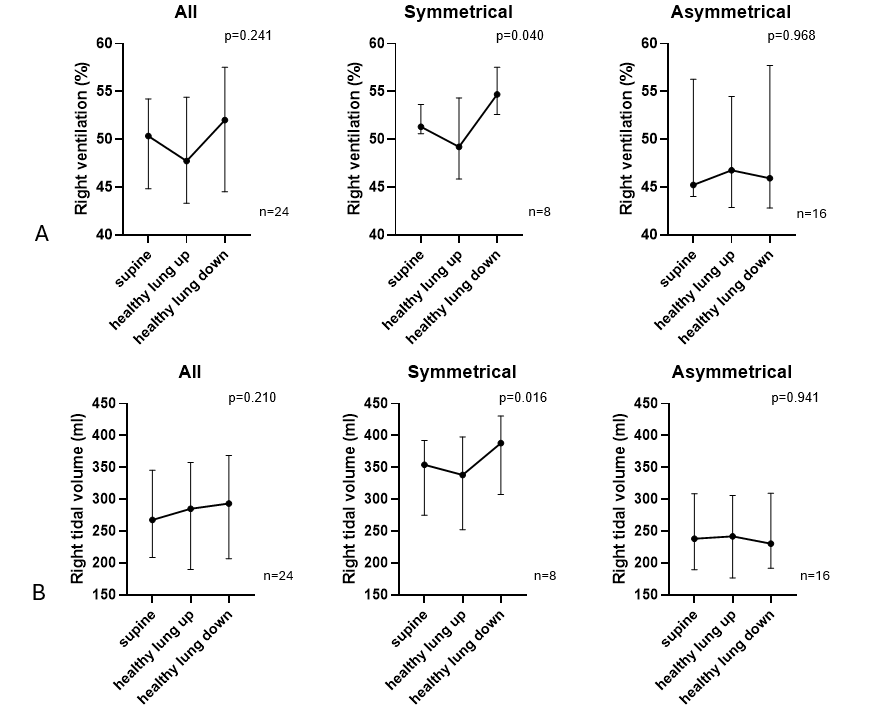


Figure E5. A. Right distribution of ventilation in percentage and B. Right volume in ml during supine, healthy lung up and healthy lung down for overall (n=24), symmetrical (n=8) and asymmetrical groups (n=16).
